# A Quantitative Method for the Characterization of Lytic Metastases of the Bone from Radiographic Images

**DOI:** 10.1155/2014/264836

**Published:** 2014-06-25

**Authors:** Alicia Baltasar Sánchez, Angel Gonzalez Sistal

**Affiliations:** Medical Imaging Research Laboratory, Department of Physiological Sciences II, Faculty of Medicine, University of Barcelona, C/ Feixa Llarga s/n Pavelló de Govern, Lab. 41.57, Hospitalet de Llobregat, 08907 Barcelona, Spain

## Abstract

The aim of our study was to assess the diagnostic usefulness of the gray level parameters to distinguish osteolytic lesions using radiological images.* Materials and Methods*. A retrospective study was carried out. A total of 76 skeletal radiographs of osteolytic metastases and 67 radiographs of multiple myeloma were used. The cases were classified into nonflat (MM1 and OL1) and flat bones (MM2 and OL2). These radiological images were analyzed by using a computerized method. The parameters calculated were mean, standard deviation, and coefficient of variation (MGL, SDGL, and CVGL) based on gray level histogram analysis of a region-of-interest. Diagnostic utility was quantified by measurement of parameters on osteolytic metastases and multiple myeloma, yielding quantification of area under the receiver operating characteristic (ROC) curve (AUC).* Results*. Flat bone groups (MM2 and OL2) showed significant differences in mean values of MGL (*P* = 0.048) and SDGL (*P* = 0.003). Their corresponding values of AUC were 0.758 for MGL and 0.883 for SDGL in flat bones. In nonflat bones these gray level parameters do not show diagnostic ability.* Conclusion*. The gray level parameters MGL and SDGL show a good discriminatory diagnostic ability to distinguish between multiple myeloma and lytic metastases in flat bones.

## 1. Introduction

One of the most important first steps when evaluating a lytic bone lesion is to know the age of the patient. Some of the lytic lesions that are largely confined to certain age groups are multiple myeloma and osteolytic metastases in the middle-aged and elderly.

Multiple myeloma is a malignant tumour of plasma cells that causes widespread lytic bone damage. It is the most common primary tumour of bone and is found in the spine, skull, ribs, sternum, and pelvis but may affect any bone with hematopoietic red marrow. The average patient age is over fifty years and the male-to-female ratio is 3 : 2. The diagnosis is based on laboratory parameters in combination with bone marrow biopsy or aspiration. The radiological appearance of multiple myeloma is characterized by irregular lytic defects of different sizes. These lytic areas are often described as “punched out” and have no periosteal reaction [1]. Moreover, it is not easy to distinguish between multiple myeloma bone disease and lytic bone metastases on plain film. In staging, treatment evaluation, and prognosis of patients with multiple myeloma, detection of lytic bone lesions has critical value. Although, new imaging techniques have been introduced to assess the extent and severity of multiple myeloma, most institutions still use radiograph as a complementary technique to evaluate disease stage (progression and therapy response) [2].

Metastatic cancer is the most common malignant secondary bone tumour. Skeletal metastases are classified according to their radiologic appearance as osteolytic, mixed, or osteoblastic. Cancers that are most likely to metastasize to bone are breast, lung, prostate, thyroid, and kidney. The average patient age is over forty years. The distribution of skeletal metastases in adults is very similar to that of hematopoietic red marrow, which coincides with the trabecular and flat bones [3]. Thus, the typical radiological imaging of a lytic metastasis appears as an area of loss of mineral bone density.

Diagnosis and classification of these bone lesions are commonly made by a variety of imaging modalities, including plain radiography (XR), skeletal scintigraphy (SS), computed tomography (CT), magnetic resonance imaging (MRI), and positron emission tomography (PET) [3, 4].

X-ray is the first imaging study undertaken to detect lytic metastases and myeloma-caused bone damage to demonstrate loss or thinning of bone (osteoporosis or osteopenia), holes in bone (lytic lesions), and/or fractures. Despite low cost and wide availability, X-rays have an important limitation: 30% of the bone must be missing before damage can be revealed.

The lytic bone disease in multiple myeloma differs from that in other cancer patients who have lytic bone metastases. Although increased osteoclastic bone destruction is involved in multiple myeloma in contrast to osteolytic metastases, once the multiple myeloma tumour burden exceeds 50% in a local area, osteoclast activity is either suppressed or absent [5].

The aim of our study was to assess the diagnostic usefulness of the gray level parameters to distinguish between osteolytic metastases and multiple myeloma from radiographic images.

## 2. Materials and Methods

### 2.1. Imaging Database

The data collection was scheduled in two separate cycles. In the first cycle a set of 76 anteroposterior radiographs with confirmed osteolytic metastases (OL) as determined from ^99m^Tc-bone scintigraphy and ^18^F-FDG PET examinations were included in this study. Patients presented antecedent of adenocarcinoma of the lung with evidence of distant metastases (M1) and no prior treatment was studied. Their mean age was 61 years (range 43–81 years, 18 males and 27 females). In the second cycle a total of 67 anteroposterior radiographs with confirmed multiple myeloma (MM) as determined from ^18^F-FDG PET examination and laboratory parameters were included too. The median age was 63 years (range 51–72 years, 17 males and 14 females).

Radiographs were performed with the following settings: 70–80 kVp, 100 cm focus to film distance, and use of a fast screen and film cassette (30 cm × 40 cm).

The Institutional Review Boards of the participating center approved this retrospective study. The radiological images used in this paper were obtained from the database of “Medical Imaging Research” Laboratory at the Department of Physiological Sciences II, Faculty of Medicine, University of Barcelona. Patient confidentiality was protected.

### 2.2. Methods

In an earlier work, an image processing and analysis method was introduced in order to characterize skeletal digitized radiographs. Hence, by means of gray level parameters on digitized radiographs we classified healthy bone according to histological and anatomical features. So, we reported an optimized healthy bone classification into two groups: flat or nonflat bones (trabecular, cortical) [6].

The images were processed and characterized with a computerized method developed by our group in an earlier work [6–8]. The workflow of image processing analysis includes the following steps: (1) image acquisition, (2) selection of a region of interest (ROI), (3) filtering for noise reduction, (4) gray level histogram (parameters output), and (5) statistical analysis to distinguish between groups.

The radiographs were digitized by using a laser scanner (KFDR-S; Konica, Tokyo, Japan) with a 0.175 mm pixel size, a matrix size of 2,048 × 2,048, and 12-bit gray-scale levels. Digitizer performance was evaluated employing a quality control protocol [5].

The images were processed using ImageJ software (NIH image program). The cases were obtained from region of interests of 40 × 50 pixels outlined manually on each radiograph. Only one ROI from each radiograph was used. They were classified into two groups, flat or nonflat bones, according to histological and anatomical bone features [6]. The final set contained 67 ROIs from multiple myeloma bone disease (flat bone: 36; nonflat bone: 31) and 45 ROIs from osteolytic metastases (flat bone: 41; nonflat bone: 35).

Due to the presence of intensity inhomogeneities and noise on radiographs inherent to imaging process, the ROI was subjected to an anisotropic diffusion filter [9, 10] which smoothed out the noise and preserved the edge and contrast associated with bone structure at the same time.

**Figure 1:**
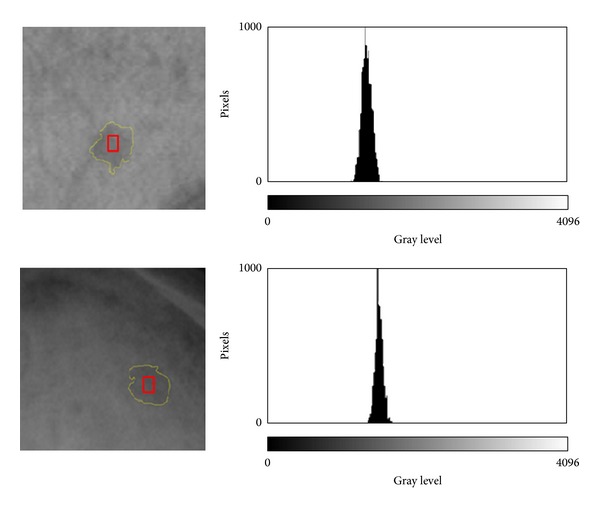
Two zooms of different plain radiographs showing osteolytic regions of interest (ROI). Gray level histogram for the outlined ROI is shown on the right side for each case. (a) Multiple myeloma (case 3, skull); (b) lytic metastasis (case 7, skull).

The parameters calculated from radiographs were based on the gray level histogram analysis of ROI (see Figure 1): mean gray level (MGL), standard deviation gray level (SDGL), and coefficient of variation (CVGL). The mean gray level is defined as the value given by the average of gray level of each ROI pixel. MGL provides 4096 gray levels because we use images of 12 bit grayscale (0–4096, where 0 is equivalent to black and 4096 to white). The standard deviation gray level of ROI pixel calculates the dispersion of gray values from the average (MGL). SDGL can be expressed in relation to MGL as a coefficient of variation (in %) and is expressed as CVGL = (SDGL/MGL) · 100.

### 2.3. Statistical Analysis

The data were analyzed using SPSS 16.0 (SPSS, Inc., Chicago, IL). Standard descriptive summary statistics was used to show overall trends in data. The data comparison among bone groups was implemented using Student's paired *t*-test. Nonparametric estimation of areas under ROC curve (AUC) was carried out to assess the diagnostic ability of each parameter considered (MGL, SDGL, and CVGL) in multiple myeloma bone disease and osteolytic metastases. Significance was considered to be reached at *P* < 0.05.

## 3. Results

Table 1 shows the descriptive statistics for mean gray level, standard deviation gray level, and coefficient of variation gray level parameters for the groups: osteolytic metastases (nonflat bone: OL1; flat bone: OL2) and multiple myeloma (nonflat bone: MM1; flat bone: MM2). When comparing the gray level parameters between nonflat bone groups (MM1 and OL1) there were no significant differences. In contrast, flat bone groups (MM2 and OL2) showed significant differences in mean values of MGL (*P* = 0.048) and SDGL (*P* = 0.003).

**Figure 2:**
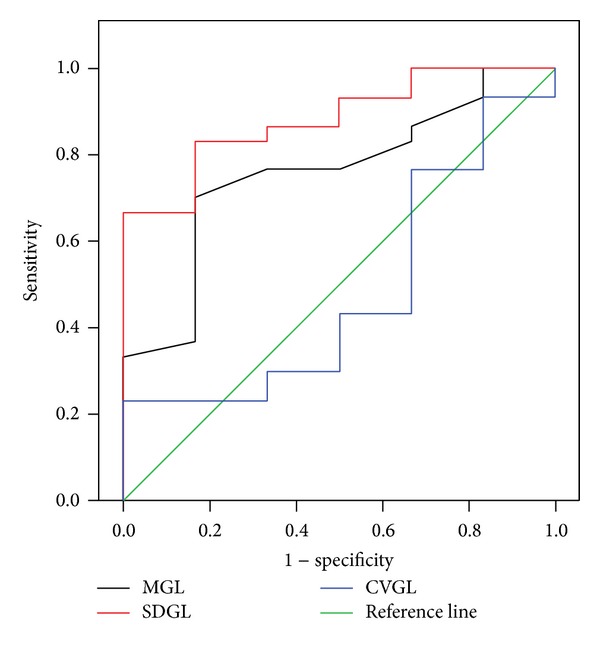
ROC curve for the three parameters considered (mean (MGL), standard deviation (SDGL), and coefficient of variation of gray level (CVGL)) when comparing flat bone groups (multiple myeloma MM2 versus osteolytic metastases OL2).

Table 2 shows the AUC values for the groups studied. There were significant values of AUC when comparing flat bone groups of multiple myeloma and osteolytic metastases (MM2 and OL2) for the MGL and SDGL parameters (AUC values: MGL = 0.758; SDGL = 0.883). These results are illustrated in Figure 2: AUC values correspond to the ROC curve when comparing gray level parameters for flat bone groups. Nevertheless, when comparing nonflat bone groups (MM1 and OL1) there were no significant values of AUC for gray level parameters.

## 4. Discussion

This study seeks to evaluate the diagnostic accuracy of gray level parameters to distinguish osteolytic lesions from two different pathologies (metastases and multiple myeloma) using radiographs.

As for nonflat bones, gray level parameters were not able to distinguish between multiple myeloma and osteolytic metastases groups.

As regards flat bones, multiple myeloma had gray levels lower than those of lytic metastases for MGL and SDGL parameters (*P* = 0.048 and *P* = 0.003, resp.). In contrast, the CVGL parameter was not able to distinguish between these groups (*P* = 0.89). When comparing multiple myeloma and osteolytic metastases, SDGL proved to have the best discriminatory ability (AUC = 0.883) and MGL a good discriminatory ability (AUC = 0.758). This is important for establishing the differential diagnosis in the two groups because the distribution of skeletal metastases and multiple myeloma bone disease is closely related to the location of the flat bones (e.g., skull, ribs, sternum, and pelvis). Histologically flat bone is made up of the two cortical thin sheets, involving a small proportion of trabecular tissue (diploe: soft spongy material containing bone marrow). The finding that myeloma lesions in flat bones appear to manifest lower gray level values might be explained as follows: in multiple myeloma, osteoclasts accumulate only at bone resorbing surfaces adjacent to myeloma cells; their levels are not increased in areas uninvolved with tumor. In addition to the increase in bone resorption, bone formation is suppressed so that bone lesions in patients with myeloma become purely lytic (there is no osteoblastic response). In osteolytic metastases, the mechanisms responsible for tumor growth in bone are complex and involve tumor stimulation of the osteoclast and the osteoblast as well as the response of the bone microenvironment [11].

There are currently different imaging modalities (plain radiography, skeletal scintigraphy, computed tomography, magnetic resonance imaging, and positron emission tomography) to diagnose multiple myeloma bone disease or lytic metastases. An accurate assessment of the response of both pathologies to treatment requires the structural changes in the bone to be visualized. In this regard, each of the aforementioned imaging techniques has its pros and cons [12–15]. Nowadays, the baseline diagnosis evaluation to detect lytic bone lesions comprises conventional radiography too [6, 9, 10, 15]. Early identification of direct anatomic visualization of the bone or tumor could lead to changes in patient management and quality of life. Although bone metastases can be treated, their response to treatment is considered “unmeasurable,” which excludes patients with cancer and bone metastatic disease from participating in clinical trials of new treatments [14]. Radiography is commonly used to evaluate symptomatic sites and is a useful complement to scintigraphy for clarifying nonspecific or atypical findings or for following up cases in which clinical findings indicate bone pain but where scintigraphy findings are negative.

The accurate detection of lytic bone lesions should improve by quantifying these lesions, thereby paving the way for computerized methods that would enable us to quantify the selected regions in order to reduce subjectivity in the interpretation of the image, calculate the ideal parameters, define patterns of normality, and determine the pathology by evaluating deviations of these indexes. Moreover, this digital method can be useful to study the evolution of these lytic bone lesions under treatment, to recognize new lesions, and to differentiate it from previous lesions.

The advantages of this methodology are its wide diffusion, low cost, and improved patient comfort.

This methodology could be applied to questions of clinical relevance. For example, bisphosphonates are administered as a preventive treatment of bone complications encountered in multiple myeloma and osteolytic metastases. However, in recent years a relationship has been established between these drugs and a new bone injury: jaw osteonecrosis [11]. This lesion is characterized by avascular necrosis of bone that was isolated from the jaws. This methodology offers the possibility of studying radiological manifestations of this disease.

This study determined preliminary results about the role of gray level image parameters on digitized radiograph in quantifying and differentiating the two bone diseases. Consequently, our results demonstrate that gray level parameters quantify multiple myeloma and lytic metastases bone lesions in flat bones accurately. This can be helpful as a complementary method for differential diagnosis. Most cases (80–90% approximately) of bone metastases and multiple myeloma bone lesions are located in the axial skeleton (spine, ribs, skull, femur, and pelvis), which are mainly flat bones.

In conclusion, the gray level parameters MGL and SDGL show a good discriminatory diagnostic ability to distinguish between multiple myeloma and lytic metastases in flat bones (AUC = 0.758 and 0.883, resp.).

## Figures and Tables

**Table 1:** Descriptive statistics for the three parameters studied: mean (MGL), standard deviation (SDGL), and coefficient of variation (CVGL) of gray level.

	Groups	Mean	St. dev.	Min.	Max.
MGL	MM1	1710.42	332.25	1264	2017
	OL1	1634.13	269.36	1312	2000
	MM2	1593.21	140.87	1406	1840
	OL2	1744.53	176.75	1472	2096

SDGL	MM1	256.89	12.98	242.37	277.56
	OL1	258.56	20.43	228.64	295.52
	MM2	248.12	9.51	238.10	264.27
	OL2	270.66	16.80	240.64	301.28

CVGL	MM1	15.02	3.09	12.64	20.43
	OL1	16.14	2.41	12.85	20.34
	MM2	15.57	1.45	13.23	16.87
	OL2	15.64	1.56	12.02	18.61

*Note.* Multiple myeloma: MM1 (nonflat bone) and MM2 (flat bone); osteolytic metastases: OL1 (nonflat bone) and OL2 (flat bone).

**Table 2:** AUC values of the ROC curve for the three parameters (mean (MGL), standard deviation (SDGL), and coefficient of variation (CVGL) of gray level) considered and their corresponding significance. Null hypothesis tested (AUC = 0.5) corresponds to a null diagnostic value to differentiate between multiple myeloma and osteolytic metastases groups.

AUC values			
Groups	MGL	SDGL	CVGL
MM1—OL1	0.420 (*P* = 0.60)	0.467 (*P* = 0.83)	0.600 (*P* = 0.52)
MM2—OL2	0.758 (*P* = 0.048)	0.883 (*P* = 0.003)	0.483 (*P* = 0.89)

*Note.* Multiple myeloma: MM1 (nonflat bone) and MM2 (flat bone); osteolytic metastases: OL1 (nonflat bone) and OL2 (flat bone). AUC: the area under the ROC curve.
